# Long non-coding RNA XLOC_000647 suppresses progression of pancreatic cancer and decreases epithelial-mesenchymal transition-induced cell invasion by down-regulating NLRP3

**DOI:** 10.1186/s12943-018-0761-9

**Published:** 2018-01-31

**Authors:** Hao Hu, Yandong Wang, Xiangya Ding, Yuan He, Zipeng Lu, Pengfei Wu, Lei Tian, Hao Yuan, Dongfang Liu, Guodong Shi, Tianfang Xia, Jie Yin, Baobao Cai, Yi Miao, Kuirong Jiang

**Affiliations:** 1 Pancreas Center, Nanjing Medical University, 300 Guangzhou Rd, Gulou District, Nanjing, Jiangsu Province 210029 China; 2 Department of Hepatopancreatobiliary Center, The Third Hospital Affiliated to Nantong University, Wuxi, 214041 China; 3 Department of General Surgery, The Second People’s Hospital of Wuhu, Wuhu, 241000 China; 4 Department of Microbiology, Nanjing Medical University, Nanjing, 211166 China; 5 Department of General Surgery, Huai’an Hospital Affiliated to Xuzhou Medical University, Huai’an, 223001 China; 6 Pancreas Institute, Nanjing Medical University, Nanjing, 210029 China; 7 Department of General Surgery, Huai’an First Hospital Affiliated to Nanjing Medical University, Huai’an, 223001 China

**Keywords:** LncRNAs, Pancreatic cancer, Invasion, Epithelial mesenchymal transition, NLRP3

## Abstract

**Background:**

Long non-coding RNAs (lncRNAs) play an important role in the development and progression of various tumors, including pancreatic cancer (PC). Recent studies have shown that lncRNAs can ‘act in cis’ to regulate the expression of its neighboring genes. Previously, we used lncRNAs microarray to identify a novel lncRNA termed XLOC_000647 that was down-regulated in PC tissues. However, the expression and function of XLOC_000647 in PC remain unclear.

**Methods:**

The expression of XLOC_000647 and NLRP3 in PC specimens and cell lines were detected by quantitative real-time PCR. Transwell assays were used to determine migration and invasion of PC cells. Western blot was carried out for detection of epithelial-mesenchymal transition (EMT) markers in PC cells. The effect of XLOC_000647 on PC cells was assessed in vitro and in vivo. The function of NOD-like receptor family pyrin domain-containing 3 (NLRP3) in PC was investigated in vitro. In addition, the regulation of NLRP3 by XLOC_000647 in PC was examined in vitro.

**Results:**

Here, XLOC_000647 expression was down-regulated in PC tissues and cell lines. The expression level of XLOC_000647 was significantly correlated to tumor stage, lymph node metastasis, and overall survival. Overexpression of XLOC_000647 attenuated cell proliferation, invasion, and EMT in vitro and impaired tumor growth in vivo. Further, a significantly negative correlation was observed between XLOC_000647 levels and its genomic nearby gene NLRP3 in vitro and in vivo. Moreover, XLOC_000647 decreased NLRP3 by inhibiting its promoter activity. Knockdown of NLRP3 decreased proliferation of cancer cells, invasion, and EMT in vitro. Importantly, after XLOC_000647 was overexpressed, the corresponding phenotypes of cells invasion and EMT were reversed by overexpression of NLRP3.

**Conclusions:**

Together, these results indicate that XLOC_000647 functions as a novel tumor suppressor of lncRNA and acts as an important regulator of NLRP3, inhibiting cell proliferation, invasion, and EMT in PC.

## Background

Pancreatic cancer (PC) is one of most aggressive and lethal malignancies with a 5-year survival rate of <7% [1, 2]. The poor overall prognosis of PC is mainly due to early distant metastasis, late diagnosis, and ineffective treatment regimens [3, 4]. Although PC research has been a focus for scientists during the past few decades, survival rates remain dismal [2, 4]. Therefore, revealing novel therapeutic targets and treatment options has become an urgent problem that needs to be addressed.

Long non-coding RNAs (lncRNAs), currently defined as transcripts of greater than 200 nucleotides without evident protein coding function [5], have been shown to play essential roles in diverse biological processes, such as epigenetic regulation, chromatin remodeling, alternative splicing, and gene expression regulation [6, 7]. Recent studies have implicated that lncRNAs not only emerge as regulators of normal cell development [8, 9], but are also involved in the development and progression of cancers [10, 11]. Dysregulation of lncRNAs has been demonstrated in breast cancer [12], gastric cancer [13], colorectal cancer [14], cholangiocarcinoma [15], hepatocellular carcinoma [16], and PC [17, 18] affecting cellular functions such as cell proliferation, migration, invasion, promotion of tumor growth, and metastasis. In our previous study, we used tissue microarrays to explore the differential expression profiles of lncRNAs and mRNAs in PC. A large number of differentially expressed lncRNAs and mRNAs were found between PC and adjacent tissues, among which a novel lncRNA XLOC_000647 was remarkably down-regulated in PC tissues. However, the biological function of XLOC_000647 in PC has not been investigated so far.

In the present study, we assessed the expression levels of XLOC_000647 in PC tissues and cell lines, and its clinical significance was also evaluated. Then we examined the role of XLOC_000647 on PC cell proliferation, invasion, and epithelial-mesenchymal transition (EMT) on xenografted mouse models. Finally, we explored the possible molecular mechanism of XLOC_000647 in tumor invasion, shedding new light for a potential novel therapeutic target and prognostic value in PC.

## Methods

### Microarray analysis

Three pairs of PC and adjacent tissues were selected for lncRNAs microarray detection. The patients enrolled did not receive any treatment before surgery, and had no underlying diseases such as diabetes and hypertension. lncRNAs microarray detection (H1602063, Arraystar_Human_LncRNA_8x60k v3.0 1-color) was performed and analyzed by KangChen Bio-tech Inc. (Shanghai, China), among which 2074 up-regulated and 2693 down-regulated of lncRNAs were found.

### Patients and tissue samples

We collected PC and corresponding adjacent normal tissue samples from 48 patients who underwent pancreatic resection at the Pancreas Center, The First Hospital Affiliated to Nanjing Medical University, from Dec 2015 to Aug 2016. The protocol was approved by the Hospital Ethics Committee and all patients signed a written informed consent form before specimen collection. All PC and matched non-tumor specimens were diagnosed by pathology. None of the patients received radiotherapy and/or chemotherapy before surgery. The resected specimens were immediately frozen by liquid nitrogen until further use. The clinicopathologic characteristics of patients are detailed in Table 1. Follow-up data were collected for all subjects and the overall survival time was calculated from the date of surgery to the date of death or the end of follow-up (Jun 2017).

**Table 1:** Correlation between XLOC_000647 expression and clinicopathologics of PC patients^a^

Characteristics	N of cases	XLOC_000647 level		*P*-value
		Low	High	
Total cases	48	24	24	
Gender				0.365
Male	31	17	14	
Female	17	7	10	
Age				0.763
< 60	17	9	8	
≥ 60	31	15	16	
TNM stage(AJCC)^b^				0.003^**^
I	19	4	15	
II	18	11	7	
III	11	9	2	
T stage				0.454
T1	11	4	7	
T2	30	15	15	
T3	5	4	1	
T4	2	1	1	
Lymph node metastasis				0.003^**^
N0	23	6	17	
N1	16	10	6	
N2	9	8	1	

Abbreviations: *N of cases* number of cases, *TNM* Tumor node metastasis, *T stage* Tumor stage

^a^Chi-square test, ^**^P < 0.01

^b^American Joint Committee on Cancer (AJCC), patients were staged in accordance with the 8th Edition of the AJCC Cancers’ TNM Classification

### Cell culture

Human primary PC cell lines (MIA-PaCa-2, BxPC-3 and PANC-1) and human embryonic kidney cells (293 T) were purchased from American Type Culture Collection (ATCC, Manassas, USA), and the immortal human pancreatic duct epithelial cell line (HPDE6), which is considered as a normal pancreatic cell line, was obtained from Pancreas Institute, Nanjing Medical University. Cells were incubated at 37 °C in humidified air with 5% CO_2_, maintained in RPMI1640 (Gibco, CA, USA) or Dulbecco’s modified Eagle’s medium (DMEM; Gibco, CA, USA) supplemented with 10% fetal bovine serum (FBS; Gibco), 100 U/ml penicillin, and 100 mg/ml streptomycin, with or without 2.5% horse serum (Gibco).

### Construction of XLOC_000647 stable expression cell lines

For stable overexpression of XLOC_000647, the full-length XLOC_000647 cDNA was synthesized by GENEWIZ (Suzhou, China) and cloned into pBABE retroviral vector (RTV-001-PURO, Cell Biolabs, CA, USA), named as XLOC_000647-pBABE, which were confirmed by sequencing (BioSune, Shanghai, China). After transfection of Plat-A (Cell Biolabs) cells for 48 h, the retrovirus supernatants were collected. Retroviral particles were concentrated by using Retro-Concentin Virus Precipitation Solution (ExCell Bio, Shanghai, China) according to the manufacturer’s guidelines. Then cancer cells were infected with virus and polybrene overnight. Positive clones were screened with puromycin for 2–3 weeks to establish XLOC_000647 stable expression cell lines and corresponding negative control for further study.

### Knockdown or overexpression of NOD-like receptor family pyrin domain-containing 3 (NLRP3)

The expression of NLRP3 was inhibited by small hairpin RNA (shRNA) interference. shNLRP3 and its corresponding negative control pSH-U6 were purchased from Vigene Biosciences (Shandong, China). All oligonucleotide sequences were listed in Table 2. The overexpression of NLRP3 was performed by gene transfection. The full-length NLRP3 cDNA was synthesized and cloned into pENTER plasmid by Vigene Biosciences, named as NLRP3-pENTER, which were validated by sequencing.

**Table 2:** Oligonucleotide sequences for this study

Name	Sense (+)	Sequence
	Antisense (−)	
qPCR-Primer		
XLOC_000647	+	5’-GCATCCACGCCTGGTGACAAC-3′
	-	-5’-GTGACTCCGACAGCCCTTGCC-3’
NLRP3	+	5’-CTTGCATCAGTATTGAGCACCA-3′
	-	5’-CCAGTTTCTGCAGGTTACACT-3’
ACTB	+	5’-CCAACCGCGAGAAGATGACC-3′
	-	5’-AGTCCATCACGATGCCAGT-3’
shRNA		
shNLRP3	+	5’-GGATCTTCGCTGCGATCAACATTCAAGAGATGTTGATCGCAGCGAAGATCCTTTTTTA-3′
	-	5’-TAAAAAAGGATCTTCGCTGCGATCAACATCTCTTGAATGTTGATCGCAGCGAAGATCCGGA-3’
pSH-U6	+	5’-GCACCCAGTCCGCCCTGAGCAAATTCAAGAGATTTGCTCAGGGCGGACTGGGTGCTTTTT-3′
	-	5’-AAAAAGCACCCAGTCCGCCCTGAGCAAATCTCTTGAATTTGCTCAGGGCGGACTGGGTGC-3’

### Cell proliferation assay

Cell proliferation was detected using a cell counting kit-8 (CCK-8, Dojindo, Japan) following the manufacturer’s protocol. Cells were plated on 96-well plates (1 × 10^3^ cells/well) and 10 μL of CCK-8 solution was added at the right time on days1 to 5. Then, the cells were incubated for 2 h at 37 °C, and finally, the cells were determined for absorbance by a fluorescent microplate reader at 450 nm. The assays were repeated in triplicate.

### Migration and invasion assay

Cells’ migration and invasion ability were measured using transwell chambers (24-well insert, 8 μm, Millipore, USA). The chambers were coated with 1:8 diluted Matrigel Matrix (Invasion assay, Corning, USA) for 1 h at 37 °C, 5 × 10^4^ cells were placed in upper chamber with 200 μL serum-free medium. The lower chamber was filled with 600 μL of medium containing 10% FBS with or without 2.5% horse serum. After incubation for 48 h, the number of cells passing through the bottom membrane of the chamber was counted under a microscope in five random fields. For motility assays, 1 × 10^4^ cells were seeded in the upper chamber without Matrigel. Other procedures were similar to the invasion assay.

### In vivo tumor formation assay

Male athymic BALB/c nude mice, 4–6 weeks old, were purchased from Vital River Laboratory Animal Technology Co. (Beijing, China). The animal care and experimental protocols were approved by the institutional guidelines of Jiangsu Province and by the Animal Care and Use Committee of Nanjing Medical University. 1 × 10^7^ PC cells were resuspended in 200 μL PBS medium and were subcutaneously injected into the flank of each nude mouse. The tumors were measured weekly and the tumor volume was calculated following the formula length × width^2^/2. The mice were killed at 6 weeks after inoculation.

### RNA extraction and quantitative real-time PCR (qRT-PCR)

Total RNA was extracted from cells or tissues using TRIzol (Invitrogen) according to the manufacturer’s instructions. For the mRNA quantitative assay, total RNAs were reverse transcribed using the reverse transcription kit (Vazyme, China) following the manufacturer’s protocol. qPCR was performed using SYBR Green Master Mix (Vazyme, China) and analyzed on a Roche LightCycler system (Roche, Switzerland). ACTB was selected as the internal reference. All the primer sequences were listed in Table 2. The results were normalized to the expression of ACTB and showed as the fold change (2^−ΔΔCT^). To clarify the clinical significance of XLOC_000647, the tissues samples were divided into two groups (high expression group vs. low expression group) by using the median expression value of XLOC_000647. Each result was repeated three times.

### Western blot assay

Total protein was extracted from the cells using whole cell lysis assay kit (KeyGEN, Nanjing, China) according to the manufacturer’s protocol. Equivalent amounts of proteins from each sample were subjected to SDS-PAGE electrophoresis and then transferred to a PVDF membrane (Millipore), blocked in 5% fat-free milk for 1 h at room temperature, incubated with specific primary antibodies overnight at 4 °C with gentle shaking, and followed by detection with enhanced chemiluminescence system (SuperSignal West Femto trial kit, Pierce). Primary antibodies were as follows: E-cadherin (1:300, 3195, CST), Vimentin (1:1000, 5741, CST), NLRP3 (1:1000, ab16097, Abcam), and ACTB (1:5000, AT0001, CMCTAG).

### Immunohistochemistry (IHC) analysis

NLRP3 and Ki67 were detected in both human PC tissue specimens and xenograft tumor specimens from nude mice, which were fixed in 4% paraformaldehyde and subsequently embedded in paraffin. After dewaxing and hydration, antigen retrieval and blocking, the 5 μm sections were incubated with specific primary antibodies overnight at 4 °C, and followed by the observation using 3,3-diaminobenzidine color kit (Dako,Denmark). Primary antibodies were as follows: NLRP3 (1:200, ab214185, Abcam) and Ki67 (1:500, ab92742, Abcam).

### Dual-luciferase reporter assay

The dual luciferase reporter assay was employed to examine the XLOC_000647 and NLRP3 relationship. Luciferase reporter plasmid containing NLRP3 promoter sequence (NLRP3 promoter-pGL-3-Basic, referred to as NLRP3 promoter) and its corresponding negative control pGL-3-Basic were purchased from Vigene Biosciences (Shandong, China). 1 × 10^4^ 293 T cells were plated on 48-well plates with 200 μL culture medium. The target plasmid (pBABE or XLOC_000647 and pGL-3-Basic or NLRP3 promoter) and pRL-TK plasmid expressing Renilla luciferase (Promega, USA) were co-transfected into cells with 50–60% confluency. After incubation for 48 h, cells were harvested for luciferase reporter assay.

### Statistical analysis

All quantitative data were presented as mean ± SD. The chi-square test (*χ*^2^ test) and Fisher’s exact test were used for categorical variables, and Student’s *t* test or ANOVA for quantitative variables. Differences in patient survival were performed using the Kaplan-Meier method and analyzed by log-rank test. The relative risk for each factor was evaluated using univariate and multivariate Cox regression analysis. Correlation analysis was explored by Pearson’s correlation. Statistical analysis and graph presentation were performed using SPSS v.17.0 software (SPSS Inc., Chicago, IL) and GraphPad Prism 5 software (GraphPad, San Diego, CA). A *P* value of <0.05 was regarded as statistically significant.

## Results

### XLOC_000647 expression is down-regulated in PC tissues and cell lines

**Fig. 1:**
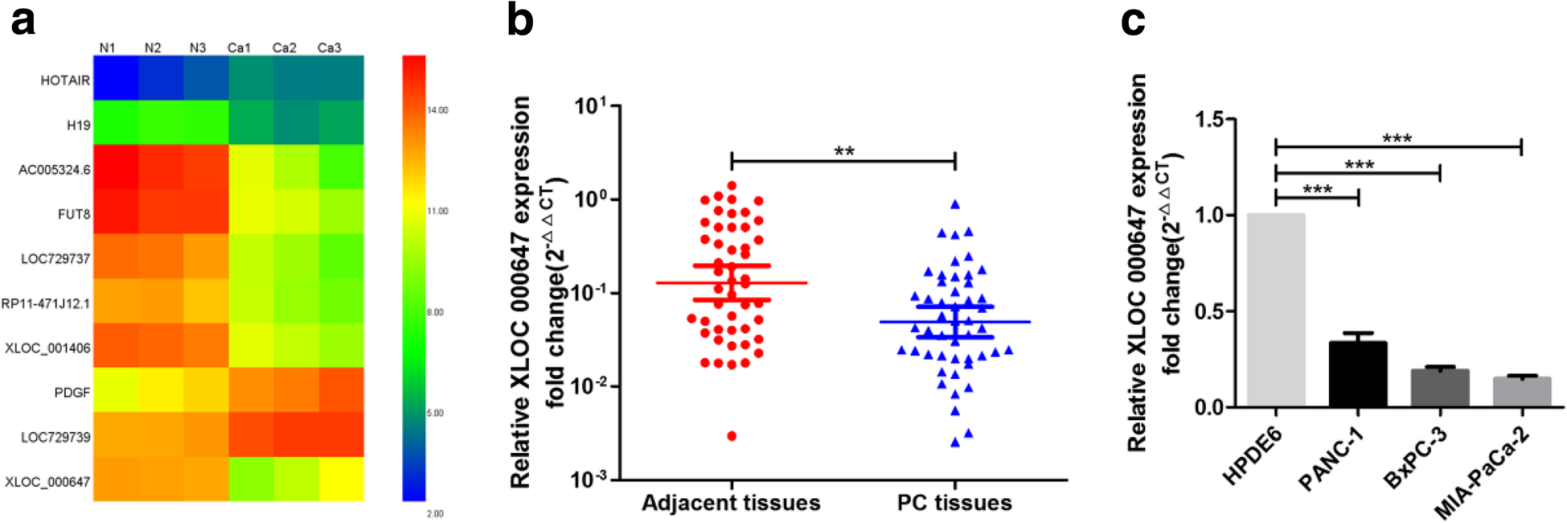
Expression of XLOC_000647 in pancreatic cancer (PC) tissues and cell lines. **a** Heat maps from our previous lncRNAs tissues microarray. N represents normal pancreatic tissue and Ca represents PC tissue. **b** Relative XLOC_000647 expression in tissues (*n* = 48). XLOC_000647 expression from all tissues was normalized to ACTB expression (ΔCT) and then compared with an adjacent tissue and converted to the fold change (2^−ΔΔCT^). **c** Relative XLOC_000647 expression in cell lines. Data are shown as fold change (2^−ΔΔCT^) and the mean ± SD from three independent experiments. ^**^P < 0.01, ^***^P < 0.001

Microarray results from our previous study determined that the expression of lncRNA XLOC_000647 was significantly decreased in PC tissues relative to adjacent tissues (Fig. 1a). In this study, the expression of XLOC_000647 was further assessed with paired PC and adjacent tissues from 48 patients by qRT-PCR. Compared with adjacent tissues, XLOC_000647 was remarkably decreased in PC tissues (Fig. 1b). And the expression level of XLOC_000647 in three PC cell lines (MIA-PaCa-2, BxPC-3, and PANC-1) was dramatically lower than in normal human pancreatic ductal epithelial cell line HPDE6 (Fig. 1c). Thus our data suggest that XLOC_000647 is decreased in PC tissues and cell lines.

### XLOC_000647 is correlated with clinicopathologic characteristics and prognosis of PC patients

**Fig. 2:**
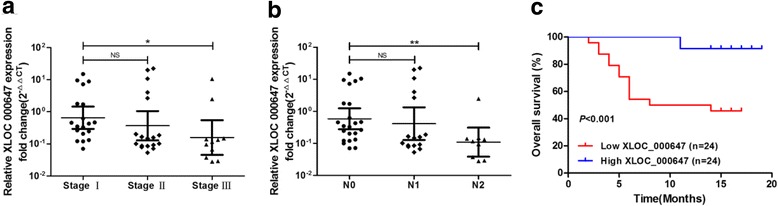
Associations between XLOC_000647, clinicopathologic characteristics and prognosis after surgery. **a** XLOC_000647 expression in different TNM stages of PC, stages I, II, and III (*n* = 19, *n* = 18 and *n* = 11, respectively). **b** XLOC_000647 expression in the lymph node metastasis- N0 group (N0, *n* = 23), -N1 group (N1, *n* = 16) and -N2 group (N2, *n* = 9). The qRT-PCR data are shown as fold change (2^−ΔΔCT^). For expressions in tissues, the levels were firstly normalized to ACTB expression as ΔCT and then compared with one of the tissues and converted to the fold change (2^−ΔΔCT^). **c** Overall survival curve of the high-level and low-level group divided by XLOC_000647 expression. The *P*-values are shown with the log-rank test (two-sided). NS (not significant). ^*^P < 0.05, ^**^P < 0.01, ^***^P < 0.001

We explored the clinical significance of XLOC_000647 in PC (Table 1). Our results showed that low expression of XLOC_000647 was significantly correlated with >advanced TNM staging, lymph node metastasis (Fig. 2a, b), and shorter survival time (log-rank test, *P* < 0.001, Fig. 2c). Moreover, the univariate analysis showed that high TNM stage, a high T stage, and a low expression of lncRNA were remarkably associated with an increased risk of cancer-related death (Table 3). Further multivariate analysis revealed that the T stage was a key prognostic factor (*P* = 0.005, Table 3). More importantly, the low expression of XLOC_000647 was found to be an independent prognostic factor associated with poor prognosis in PC (*P* = 0.004, Table 3). These data indicate that XLOC_000647 is significantly correlated with the TNM stage and lymph node metastasis and is also an independent factor for the prognosis of PC patients.

**Table 3:** Univariate and multivariate analysis of overall survival in PC patients (*n* = 48)

Variables	Univariate analysis			Multivariate analysis		
	HR	95% CI	*P*-value	HR	95% CI	*P*-value
Gender	2.063	0.706–6.025	0.186			
Age	0.470	0.161–1.367	0.166			
TNM			0.039^*^			
I vs. II	0.226	0.063–0.804	0.022^*^			
I vs. III	0.143	0.028–0.721	0.019^*^			
T stage			0.002^**^	2.699	1.351–5.389	0.005^**^
T1 vs. T2	0.405	0.096–1.711	0.219			
T1 vs. T3	0.007	0.001–0.062	0.000^***^			
T1 vs. T4	0.023	0.000–1.858	0.093			
Lymph node metastasis			0.253			
N0 vs. N1	0.490	0.142–1.689	0.259			
N0 vs. N2	0.274	0.056–1.339	0.110			0.004^**^
XLOC_000647 expression	6.759	2.368–19.290	0.000^***^	0.104	0.023–0.477	

Abbreviations: *HR* Hazard ratio; 95% CI = 95% confidence interval

Cox regression analysis, ^*^P < 0.05, ^**^P < 0.01, ^***^P < 0.001

### XLOC_000647 inhibits proliferation, invasion, and EMT of PC cells in vitro

**Fig. 3:**
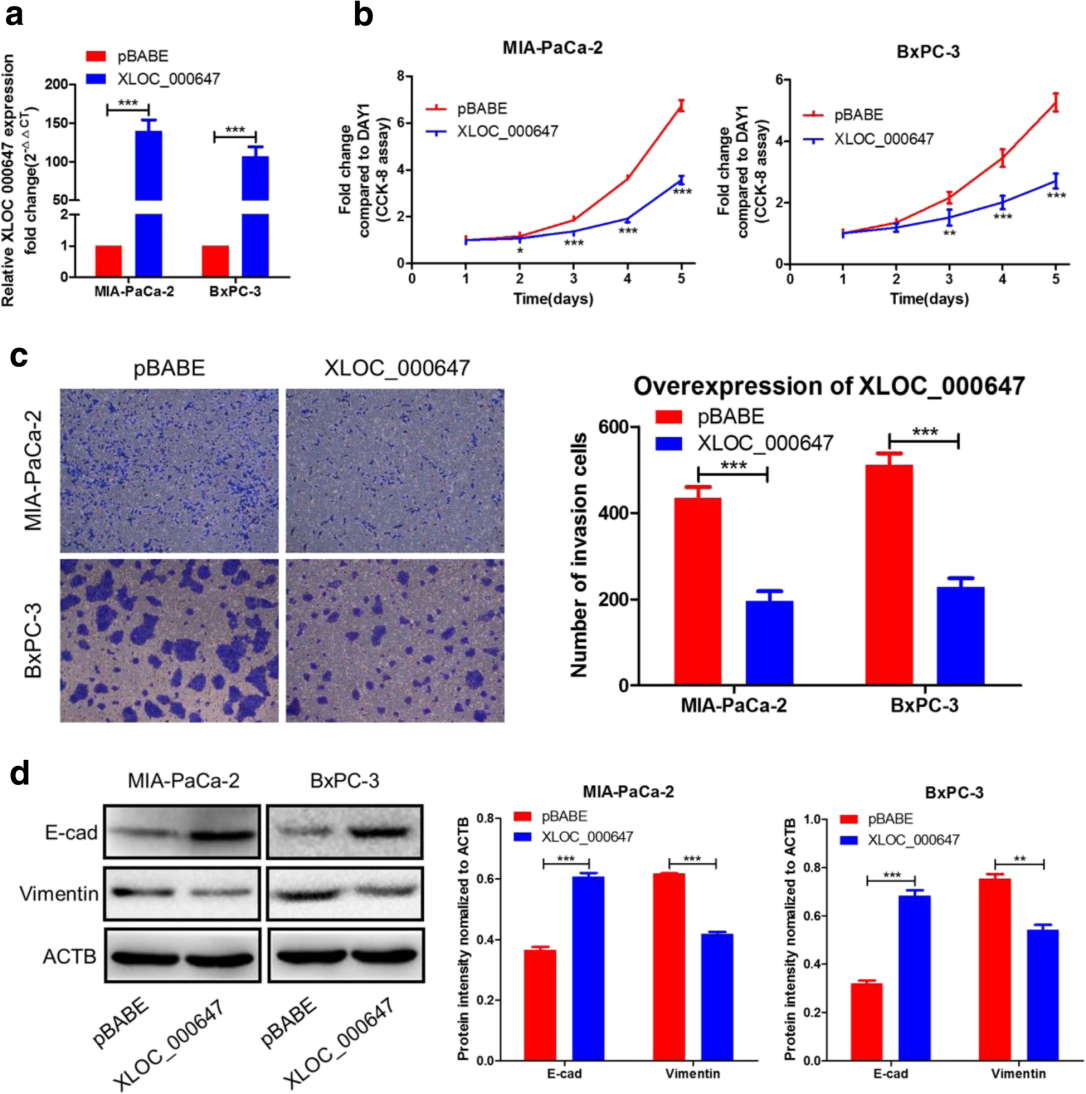
Influence of XLOC_000647 on the PC cell proliferation, cell invasion, and EMT. **a** The expression of XLOC_000647 was up-regulated by XLOC_000647-pBABE in MIA-PaCa-2 and BxPC-3 cells. **b** The proliferation activity of the two cell lines detected by CCK-8 assay. **c** The invasion capacity of the two cell lines when compared with the controls by transwell assays. **d** Analysis of the E-cad and Vimentin protein levels in the two cell lines and corresponding control cells by western blot. Results are represented as protein intensity relative to ACTB. ^*^P < 0.05, ^**^P < 0.01, ^***^P < 0.001

To investigate the effect of lncRNA on proliferation, invasion, and EMT of PC cells, we constructed a retroviral stable expression of XLOC_000647 in MIA-PaCa-2 and BxPC-3 cells. The levels of XLOC_000647 in these two cells were validated by qRT-PCR (Fig. 3a). Overexpression of XLOC_000647 decreased cell proliferation and invasion when compared with pBABE controls, as shown by the results of the CCK-8 assay and transwell chambers measurement (Fig. 3b and c). However, overexpression of XLOC_000647 had little impact on PC cells migration (data not shown). Furthermore, compared with pBABE controls, western blot results indicated that the expression of EMT-related epithelial marker E-cadherin (E-cad) was significantly increased and the mesenchymal marker Vimentin was dramatically decreased in the above two cells (Fig. 3d). These results indicate that XLOC_000647 play a key role in inhibition of proliferation and EMT-induced invasion of PC cells in vitro, but has no effect on migration of PC cells.

### XLOC_000647 suppresses tumor growth in vivo

**Fig. 4:**
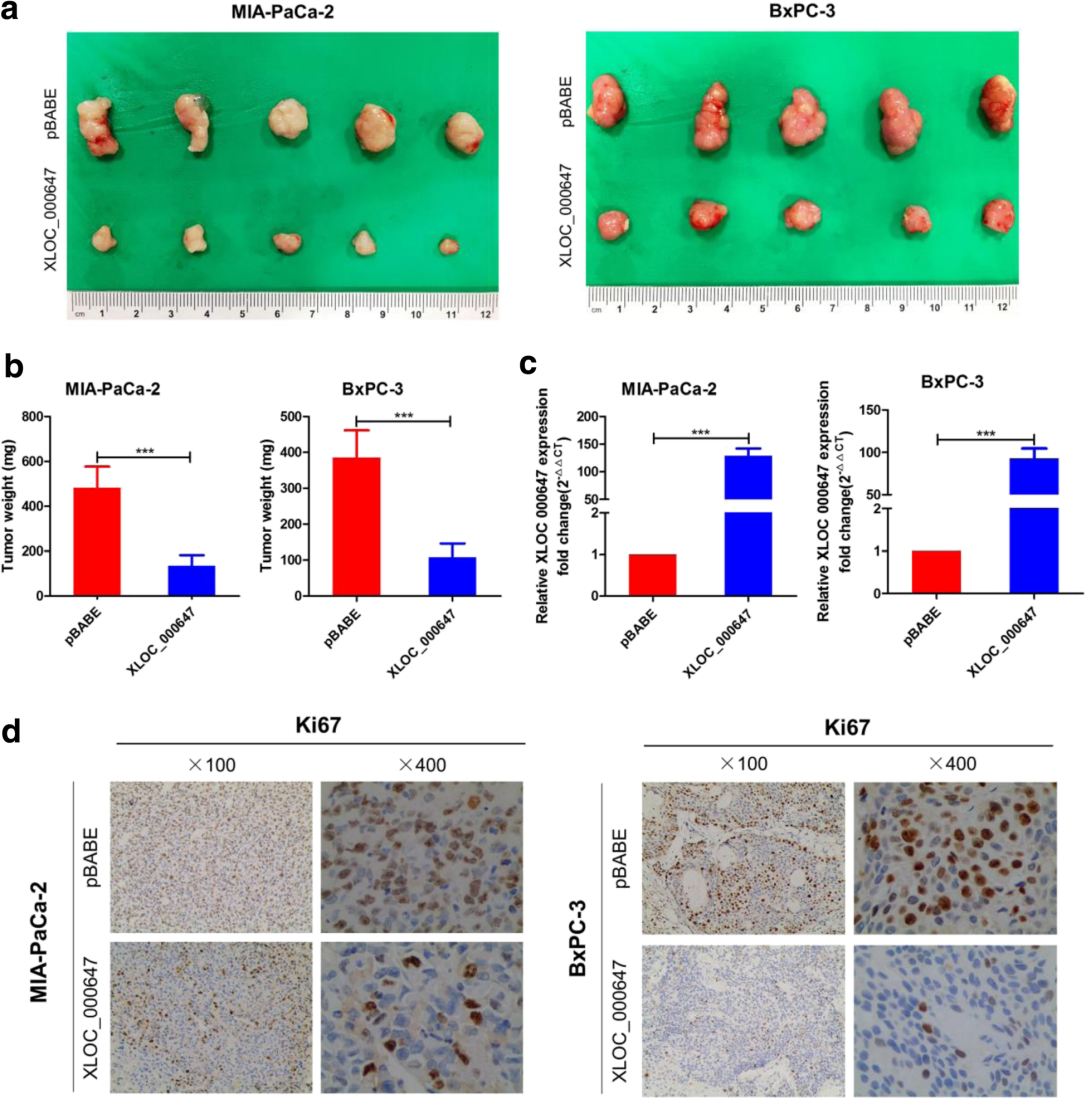
Stable overexpression of XLOC_000647 significantly reduces tumor growth in vivo. **a** Tumors removed from the mice 6 weeks after injection of MIA-PaCa-2 or BxPC-3 cells stably transfected with XLOC_000647-pBABE or pBABE, respectively. **b** Tumor weights are shown as the means ± SD when the tumors were harvested. **c** The expression of XLOC_000647 in paired tumor tissues was analyzed by qRT-PCR. **d** Representative images (×100 and ×400) of IHC staining of the tumor. Results showed that overexpression of XLOC_000647 decreased the proliferation index of Ki67. ^***^P < 0.001

We then determined the inhibition of XLOC_000647 in tumor progression in vivo. Stably expressing XLOC_000647 of MIA-PaCa-2 and BxPC-3 cells and their corresponding pBABE control cells were subcutaneously injected into nude mice. Six weeks after injection, the tumor size and weight of stably expressing XLOC_000647 groups were all remarkably lessened than the control groups (Fig. 4a and b). Moreover, we verified the inhibition of XLOC_000647 in tumors from stably expressing XLOC_000647 groups using qRT-PCR (Fig. 4c). Furthermore, immunohistochemical results of tumors showed that positive rate of cell proliferation indicator Ki67 was decreased in stably expressing XLOC_000647 groups (Fig. 4d). Taken together, our data demonstrate that XLOC_000647 acts as a tumor suppressor gene in PC tumor growth both in vitro and in vivo*.*

### NLRP3 is overexpressed in PC tissues and cell lines, which was negatively regulated by XLOC_000647

**Fig. 5:**
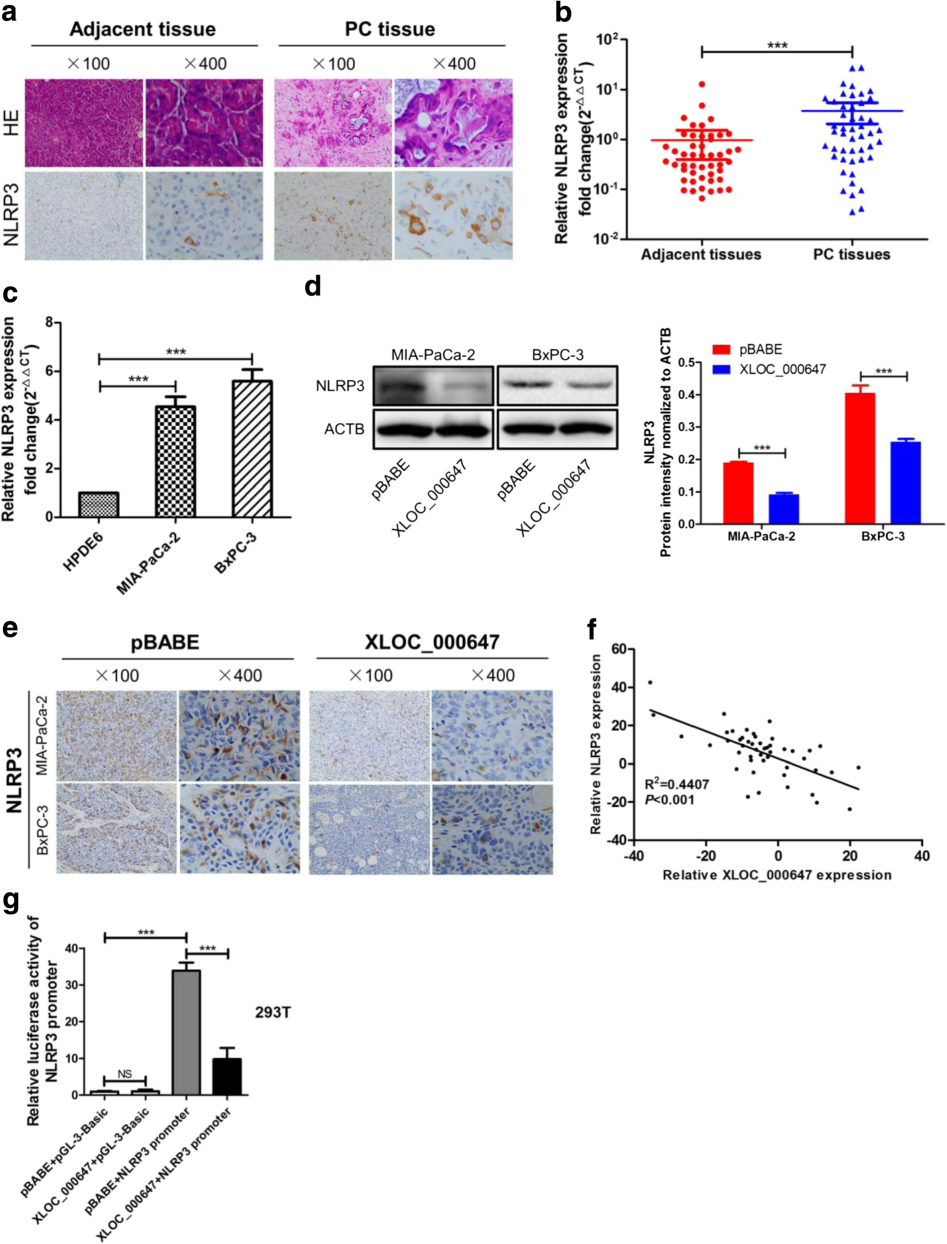
Expression of NLRP3 in PC tissues and cell lines, and association between NLRP3 and XLOC_000647. **a** Representative images (×100 and ×400) of H and E (h*ematoxylin and eosin)* and immunohistochemical (IHC) staining for NLRP3 in paraffin-embedded PC and corresponding adjacent tissues. **b** Relative XLOC_00067 expression in tissues (*n* = 48). PC tissues versus corresponding adjacent tissues. **c** Relative XLOC_000647 expression in cell lines and the mean ± SD from three independent experiments. **d** Influence of XLOC_000647-stable overexpression on the expression level of NLRP3 in cell lines by western blot. **e** Representative images (×100 and ×400) of IHC staining of the tumor from mice. Results showed that overexpression of XLOC_000647 decreased the expression level of NLRP3. **f** The correlation between NLRP3 mRNA levels and XLOC_00067 levels in 48 PC tissues (R^2^ = 0.4407, *P* < 0.001). **g** The luciferase activity of NLRP3 promoter is decreased by XLOC_000647 in 293 T cells. NS (not significant). ^***^P < 0.001

Recent studies have shown that many lncRNAs can act as local regulators to regulate the expression of its nearby genes ‘in cis’ [19–21]. Therefore, we investigated whether XLOC_000647 was involved in regulating its neighboring gene. Our microarray results showed that there are three nearby genes of lncRNA_XLOC_000647, including NLRP3, olfactory receptor family 2 subfamily B member 11 (OR2B11), zinc finger protein 496 (ZNF496). Our study mainly focused on the invasion and metastasis of pancreatic cancer. Compared with other two nearby genes, NLRP3 was reported to be involved in the metastasis of many tumors, which was in line with our research. Therefore, we finally identified NLRP3 as a candidate gene to study. NLRP3 was located at about 25 kb of the XLOC_000647 downstream. Immunohistochemistry (IHC) revealed that PC tissues exhibited an increased positive rate of NLRP3 than adjacent tissues (Fig. 5a). Then, the expression of NLRP3 was further examined with paired PC and adjacent tissues from 48 patients by qRT-PCR. Data showed NLRP3 levels of PC tissues were significantly higher than that of adjacent tissues (Fig. 5b). And similar results were detected with matched PC and HPDE6 cell lines (Fig. 5c). Furthermore, western blot and IHC results indicated that overexpression of XLOC_000647 resulted in a decreased expression of NLRP3 in PC cell lines and tumor tissues from nude mice compared with their corresponding control groups (Fig. 5d and e). More importantly, correlation analysis revealed NLRP3 mRNA levels negatively correlated with XLOC_000647 in 48 PC tissues (R^2^ = 0.4407, *P* < 0.001, Fig. 5f). And the luciferase reporter assay showed that the activity of NLRP3 promoter was greatly inhibited followed by overexpression of XLOC_000647 in 293 T cells (Fig. 5g). These results suggested that the expression of NLRP3 was negatively regulated by XLOC_000647, which mediated the proliferation, invasion, and EMT of PC.

### NLRP3 promotes proliferation, invasion, and EMT of PC cells in vitro

**Fig. 6:**
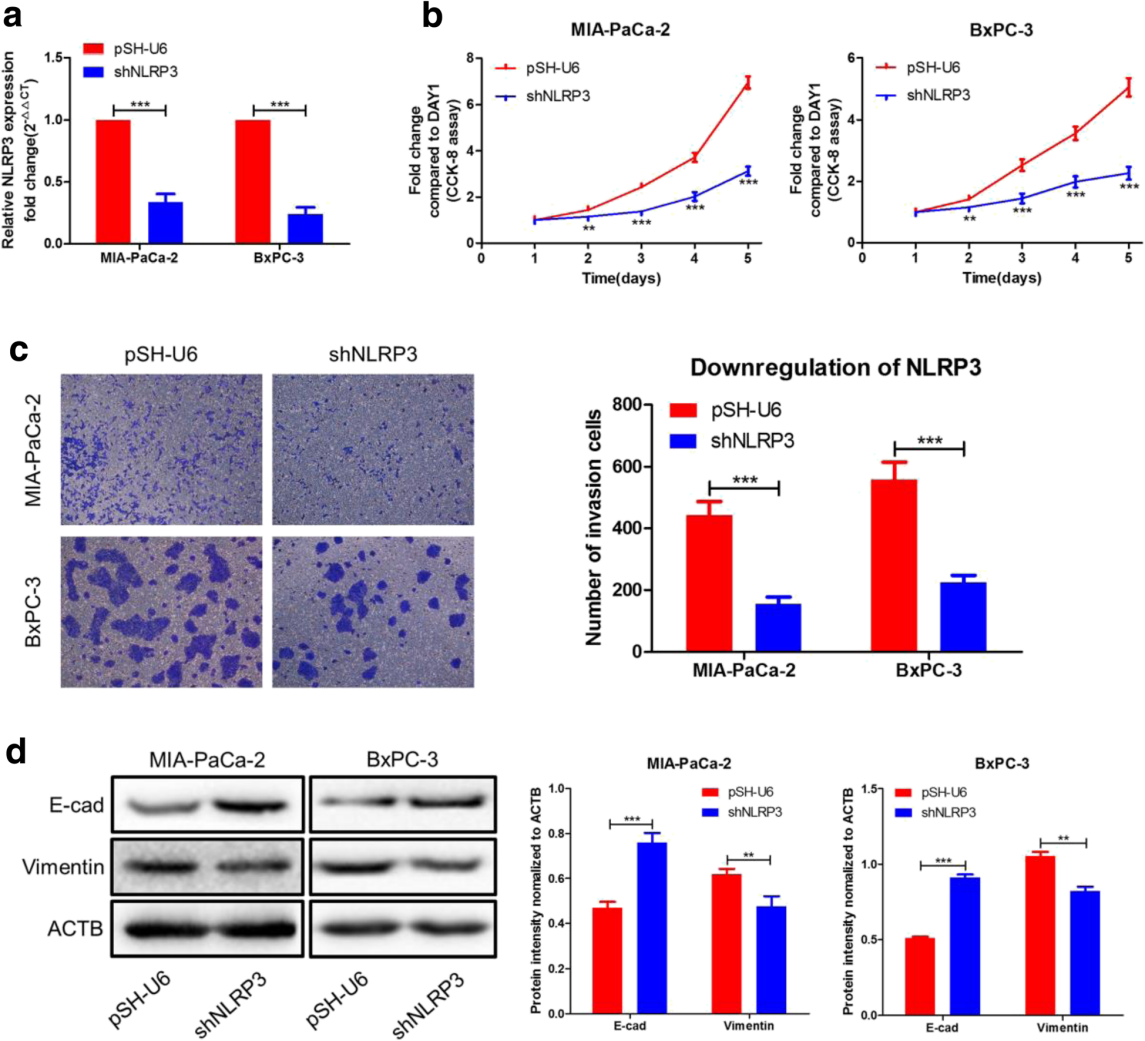
Influence of NLRP3 on PC cell proliferation, cell invasion, and EMT. **a** The expression of NLRP3 was down-regulated by shNLRP3 in MIA-PaCa-2 and BxPC-3 cells. **b** The proliferation activity of these two cell lines detected by CCK-8 assay. **c** The invasion capacity of the two cell lines when compared with the controls by transwell assays. **d** Analysis of the E-cad and Vimentin protein levels in the two cell lines and corresponding control cells by western blot. ^**^P < 0.01, ^***^P < 0.001

To explore the role of NLRP3 on proliferation, invasion, and EMT of PC, MIA-PaCa-2 and BxPC-3 cells were transfected with shNLRP3 for 48 h respectively. The levels of NLRP3 were verified by qRT-PCR (Fig. 6a). Results of CCK-8 assay and transwell chambers demonstrated that knockdown of NLRP3 suppressed cells proliferation and invasion when compared with pSH-U6 controls (Fig. 6b and c). Furthermore, compared with pSH-U6 controls, western blot showed that the expression of E-cad was remarkably up-regulated and the Vimentin was significantly down-regulated in the above two cells (Fig. 6d). These results established that NLRP3 is crucial for the proliferation, invasion, and EMT of PC cells in vitro.

### Inhibition of EMT-induced cells invasion regulated by XLOC_000647 overexpression was reversed by NLRP3 overexpression

**Fig. 7:**
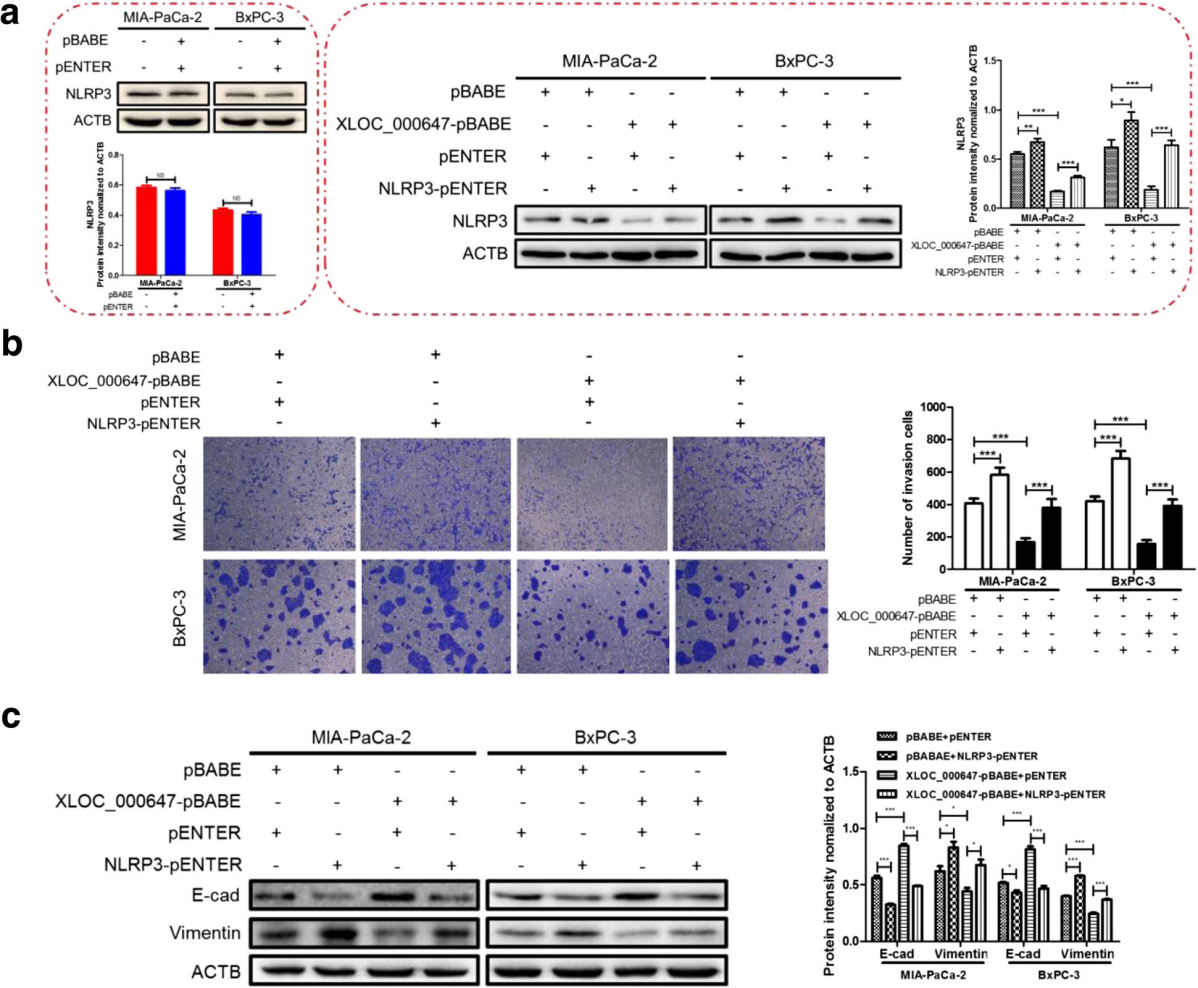
Inhibition of EMT-induced cells invasion regulated by XLOC_000647 overexpression was reversed by NLRP3 overexpression. **a** The vectors of pBABE + pENTER alone had no effect on the expression of NLRP3 (left). The protein levels of NLRP3 were up-regulated after transfected with NLRP3-pENTER in cells of XLOC_000647-stable overexpression compared with control cells (right). **b** The invasion of XLOC_000647-overexpressed cells transfected with NLRP3-pENTER when compared with the controls by transwell assays. Values represented the mean ± SD from three independent experiments. **c** Analysis of the E-cad and Vimentin protein levels in XLOC_000647-overexpressed cells transfected with NLRP3-pENTER and pENTER by western blot. NS (not significant). ^*^P < 0.05, ^**^P < 0.01, ^***^P < 0.001

Next, we investigated whether XLOC_000647 suppressed EMT induced cells invasion by down-regulating NLRP3. Firstly, stably expressing XLOC_000647 cells of MIA-PaCa-2 and BxPC-3 cells and their corresponding pBABE control cells were transfected with NLRP3-pENTER for 48 h respectively. The levels of NLRP3 were validated by western blot (Fig. 7a). The transwell assay showed that overexpression of NLRP3 restored cells invasion ability inhibited by XLOC_000647 overexpression (Fig. 7b). Moreover, compared with pENTER controls, western blot indicated that the expression of E-cad was significantly decreased and the Vimentin was remarkably increased in the above two cells of stable expressing XLOC_000647 (Fig. 7c). These data revealed that XLOC_000647 might indirectly or directly regulate the expression of NLRP3, which in turn affected EMT induced invasion of PC.

## Discussion

In the present study, we demonstrated that XLOC_000647 was down-regulated and has the potential to be clinically significant in PC. Moreover, we identified XLOC_000647 as a tumor suppressor gene in PC, XLOC_000647 overexpression in PC cells inhibited cell proliferation in vitro, and suppressed tumor formation in vivo. We also found that XLOC_000647 could reverse EMT to suppress invasion, and the genomic nearby gene NLRP3 of XLOC_000647 may present as a mediator for XLOC_000647 induced EMT reversion. These results may help us to further comprehend the molecular function of XLOC_000647 and provide novel therapeutic targets for PC.

XLOC_000647 was identified by our lncRNAs tissue microarrays of PC and corresponding adjacent tissues (H1602063, KangChen Bio-tech Inc., Shanghai, China),which derived from LincRNAs identified by Cabili et al. in 2011, was an intergenic lncRNA with 1073 nucleotides in length and encoded on the sense strand of chromosome 1. Further validation demonstrated that the expression of XLOC_000647 in PC tissues and cell lines were all reduced. Additionally, XLOC_000647 expression was correlated with the overall survival, TNM stage and lymph node metastasis of PC individuals. TNM stage and T stage were involved in cancer-related mortality. More importantly, XLOC_000647 was an independent factor for the prognosis of PC. It was noteworthy that the tumor size was also an independent risk factor for the prognosis of PC, which was consistent with Strobel and Allen’s findings [22, 23]. Although a handful of lncRNAs have been reported to be associated with the prognosis of PC [18, 24–27], to our knowledge, this was the first time XLOC_000647 was shown to be clinically related to PC.

For further confirmation and exploration of the function of XLOC_000647 in vitro and in vivo experiments were designed and carried out. We found that the overexpression of XLOC_000647 led to a decreased proliferation of PC cells in vitro and inhibition of tumor growth in vivo. Although the molecular mechanism of XLOC_000647 on cell proliferation requires an in-depth study, we identified XLOC_000647 as a tumor suppressor gene in PC. In addition, the overexpression of XLOC_000647 impaired invasion of PC cells and a low level of XLOC_000647 was associated with lymph node metastasis in PC individuals, which indicated that XLOC_000647 was involved in the metastasis of PC. Future in vivo studies aimed to investigate the inhibitory effect of XLOC_000647 on tumor metastasis are necessary. However, for this study, we mainly focused our work on the mechanism of how XLOC_000647 suppresses tumor invasion.

Invasion and metastasis are the main factors that determine the prognosis of PC [2, 28]. Furthermore, EMT has been recognized as an important event in the initiation of cancer metastasis [29, 30], during which epithelial cells lose their apical-basal polarity and develop a mesenchymal phenotype. During EMT, epithelial carcinoma cells undergo phenotypic changes that increase their motility and invasive capacities, thus facilitating their metastasis [31]. Our study revealed that overexpression of XLOC_000647 resulted in reduced invasion capacity and the reversal of EMT with increased epithelial marker E-cad and decreased mesenchymal marker Vimentin. This was the first time XLOC_000647 inhibited the invasion of PC cells by reversing EMT. Recently, the role of lncRNAs regulating the expression of its adjacent genes has been verified [19–21]. Therefore, we hypothesized that XLOC_000647 might suppress EMT by regulating its neighboring gene expression. Finally, we found a gene named NLRP3 was located at about 25 kb of XLOC_000647 downstream.

NLRP3 is a member of a nucleotide-binding domain and leucine-rich repeat-containing protein family of intracellular sensors. A previous study showed that NLRP3 forms a cytoplasmic complex called the NLRP3 inflammasome whose activation potently modulates innate immune function by regulating the maturation and secretion of inflammatory cytokines such as IL-1β and IL-18 [32, 33]. However, recent studies have confirmed that NLRP3 inflammasome with excessive activation promotes the metastasis of multiple tumors including melanoma cells and hepatocellular carcinoma cells [34–36]. Here, we noted that NLRP3 was highly expressed in PC cells and tissues, and knockdown of NLRP3 led to inhibition of cells proliferation and invasion and reversion of EMT with increased E-cad and decreased Vimentin, indicating its potentially important role in promoting PC. Additionally, overexpression of XLOC_000647 resulted in a decreased expression of NLRP3 in PC cell lines and tumor tissues from nude mice. Importantly, correlation analysis showed NLRP3 mRNA levels negatively correlated with XLOC_000647 in PC tissues. Moreover, overexpression of XLOC_000647 resulted in a decreased luciferase activity of NLRP3 promoter in vitro. Thus, we hypothesized that XLOC_000647 might play a tumor suppressor role by negative regulation of NLRP3 expression.

In order to obtain further validation, we investigated the regulatory role of XLOC_000647 on NLRP3 by in vitro experiments. We found that the protein level of NLRP3 was significantly increased after overexpression of NLRP3 in two PC cell lines stably expressing XLOC_000647. Meanwhile, the invasion capacity of these two cell lines was restored. Furthermore, EMT induced by XLOC_000647 was also reversed. Together, these results indicate that XLOC_000647 decreases EMT-induced cell invasion by down-regulating NLRP3, which are consistent with previous studies that reported NLRP3 is involved in the regulation of EMT and in turn promotes the metastasis of colon cancer and lung adenocarcinoma [37, 38]. Nevertheless, the molecular mechanism of how XLOC_000647 regulates EMT needs to be further defined.

## Conclusions

Our work was the first study to explore the regulatory role and potential molecular mechanisms of lncRNA XLOC_000647 in PC progression, cell invasion, and EMT. The results indicated that XLOC_000647 was a novel potential tumor suppressor gene in PC, and was down-regulated in PC tissues and cell lines. XLOC_000647 can suppress the progression of PC both in vitro and in vivo, and inhibit EMT-induced cell invasion by down-regulating NLRP3. We surmised that XLOC_000647 might be a potential target for therapies based on NLRP3 for the treatment of PC.

## Abbreviations

CCK-8: Cell counting kit-8
E-cad: E-cadherin
EMT: Epithelial-mesenchymal transition
HPDE6: Pancreatic duct epithelial cell line
IHC: Immunohistochemistry
lncRNAs: Long non-coding RNAs
NLRP3: NOD-like receptor family pyrin domain-containing 3
PC: Pancreatic cancer
qRT-PCR: Quantitative real-time PCR
shRNA: Small hairpin RNA
TNM: Tumor node metastasis

## References

[1] Waddell N, Pajic M, Patch AM, Chang DK, Kassahn KS, Bailey P, Johns AL, Miller D, Nones K, Quek K (2015). Whole genomes redefine the mutational landscape of pancreatic cancer. Nature.

[2] Hessmann E, Johnsen SA, Siveke JT, Ellenrieder V (2017). Epigenetic treatment of pancreatic cancer: is there a therapeutic perspective on the horizon?. Gut.

[3] Zhan HX, JW X, Wu D, Zhang TP, SY H (2015). Pancreatic cancer stem cells: new insight into a stubborn disease. Cancer Lett.

[4] Ansari D, Tingstedt B, Andersson B, Holmquist F, Sturesson C, Williamsson C, Sasor A, Borg D, Bauden M, Andersson R (2016). Pancreatic cancer: yesterday, today and tomorrow. Future Oncol.

[5] Rinn JL, Chang HY (2012). Genome regulation by long noncoding RNAs. Annu Rev Biochem.

[6] Batista PJ, Chang HY (2013). Long noncoding RNAs: cellular address codes in development and disease. Cell.

[7] Mercer TR, Mattick JS (2013). Structure and function of long noncoding RNAs in epigenetic regulation. Nat Struct Mol Biol.

[8] Arnes L, Sussel L (2015). Epigenetic modifications and long noncoding RNAs influence pancreas development and function. Trends Genet.

[9] Elling R, Chan J, Fitzgerald KA (2016). Emerging role of long noncoding RNAs as regulators of innate immune cell development and inflammatory gene expression. Eur J Immunol.

[10] Kwok ZH, Tay Y (2017). Long noncoding RNAs: lincs between human health and disease. Biochem Soc Trans.

[11] Peng WX, Koirala P, Mo YY (2017). LncRNA-mediated regulation of cell signaling in cancer. Oncogene.

[12] Fang Y, Wang J, Wu F, Song Y, Zhao S, Zhang Q (2017). Long non-coding RNA HOXA-AS2 promotes proliferation and invasion of breast cancer by acting as a miR-520c-3p sponge. Oncotarget.

[13] CE H, PZ D, Zhang HD, Huang GJ (2017). Long Noncoding RNA CRNDE Promotes proliferation of gastric cancer cells by targeting miR-145. Cell Physiol Biochem.

[14] Rigoutsos I, Lee SK, Nam SY, Anfossi S, Pasculli B, Pichler M, Jing Y, Rodriguez-Aguayo C, Telonis AG, Rossi S (2017). N-BLR, a primate-specific non-coding transcript leads to colorectal cancer invasion and migration. Genome Biol.

[15] Lu X, Zhou C, Li R, Deng Y, Zhao L, Zhai W (2017). Long Noncoding RNA AFAP1-AS1 Promoted Tumor Growth and Invasion in Cholangiocarcinoma. Cell Physiol Biochem.

[16] Wang Y, Liu Z, Yao B, Li Q, Wang L, Wang C, Dou C, Xu M, Liu Q, Tu K (2017). Long non-coding RNA CASC2 suppresses epithelial-mesenchymal transition of hepatocellular carcinoma cells through CASC2/miR-367/FBXW7 axis. Mol Cancer.

[17] Zhan HX, Wang Y, Li C, JW X, Zhou B, Zhu JK, Han HF, Wang L, Wang YS, SY H (2016). LincRNA-ROR promotes invasion, metastasis and tumor growth in pancreatic cancer through activating ZEB1 pathway. Cancer Lett.

[18] Zheng S, Chen H, Wang Y, Gao W, Fu Z, Zhou Q, Jiang Y, Lin Q, Tan L, Ye H (2016). Long non-coding RNA LOC389641 promotes progression of pancreatic ductal adenocarcinoma and increases cell invasion by regulating E-cadherin in a TNFRSF10A-related manner. Cancer Lett.

[19] Orom UA, Derrien T, Beringer M, Gumireddy K, Gardini A, Bussotti G, Lai F, Zytnicki M, Notredame C, Huang Q (2010). Long noncoding RNAs with enhancer-like function in human cells. Cell.

[20] Luo S, JY L, Liu L, Yin Y, Chen C, Han X, Wu B, Xu R, Liu W, Yan P (2016). Divergent lncRNAs regulate gene expression and lineage differentiation in pluripotent cells. Cell Stem Cell.

[21] Engreitz JM, Haines JE, Perez EM, Munson G, Chen J, Kane M, McDonel PE, Guttman M, Lander ES (2016). Local regulation of gene expression by lncRNA promoters, transcription and splicing. Nature.

[22] Strobel O, Hinz U, Gluth A, Hank T, Hackert T, Bergmann F, Werner J, Buchler MW (2015). Pancreatic adenocarcinoma: number of positive nodes allows to distinguish several N categories. Ann Surg.

[23] Allen PJ, Kuk D, Castillo CF, Basturk O, Wolfgang CL, Cameron JL, Lillemoe KD, Ferrone CR, Morales-Oyarvide V, He J (2017). Multi-institutional validation study of the American joint commission on cancer (8th edition) changes for T and N staging in patients with pancreatic adenocarcinoma. Ann Surg.

[24] Li J, Liu D, Hua R, Zhang J, Liu W, Huo Y, Cheng Y, Hong J, Sun Y (2014). Long non-coding RNAs expressed in pancreatic ductal adenocarcinoma and lncRNA BC008363 an independent prognostic factor in PDAC. Pancreatology.

[25] Qu S, Yang X, Song W, Sun W, Li X, Wang J, Zhong Y, Shang R, Ruan B, Zhang Z (2016). Downregulation of lncRNA-ATB correlates with clinical progression and unfavorable prognosis in pancreatic cancer. Tumour Biol.

[26] Gao S, Wang P, Hua Y, Xi H, Meng Z, Liu T, Chen Z, Liu L (2016). ROR functions as a ceRNA to regulate Nanog expression by sponging miR-145 and predicts poor prognosis in pancreatic cancer. Oncotarget.

[27] Li L, Zhang GQ, Chen H, Zhao ZJ, Chen HZ, Liu H, Wang G, Jia YH, Pan SH, Kong R (2016). Plasma and tumor levels of Linc-pint are diagnostic and prognostic biomarkers for pancreatic cancer. Oncotarget.

[28] Ying H, Dey P, Yao W, Kimmelman AC, Draetta GF, Maitra A, DePinho RA (2016). Genetics and biology of pancreatic ductal adenocarcinoma. Genes Dev.

[29] De Craene B, Berx G (2013). Regulatory networks defining EMT during cancer initiation and progression. Nat Rev Cancer.

[30] Yang G, Liang Y, Zheng T, Song R, Wang J, Shi H, Sun B, Xie C, Li Y, Han J (2016). FCN2 inhibits epithelial-mesenchymal transition-induced metastasis of hepatocellular carcinoma via TGF-beta/Smad signaling. Cancer Lett.

[31] Kalluri R (2009). EMT: when epithelial cells decide to become mesenchymal-like cells. J Clin Invest.

[32] Schroder K, Tschopp J (2010). The inflammasomes. Cell.

[33] Tschopp J, Schroder K (2010). NLRP3 inflammasome activation: the convergence of multiple signalling pathways on ROS production?. Nat Rev Immunol.

[34] Ahmad I, Muneer KM, Tamimi IA, Chang ME, Ata MO, Yusuf N (2013). Thymoquinone suppresses metastasis of melanoma cells by inhibition of NLRP3 inflammasome. Toxicol Appl Pharmacol.

[35] Fan SH, Wang YY, Lu J, Zheng YL, DM W, Li MQ, Hu B, Zhang ZF, Cheng W, Shan Q (2014). Luteoloside suppresses proliferation and metastasis of hepatocellular carcinoma cells by inhibition of NLRP3 inflammasome. PLoS One.

[36] Karki R, Man SM, Kanneganti TD (2017). Inflammasomes and cancer. Cancer Immunol Res.

[37] Wang H, Wang Y, Du Q, Lu P, Fan H, Lu J, Hu R (2016). Inflammasome-independent NLRP3 is required for epithelial-mesenchymal transition in colon cancer cells. Exp Cell Res.

[38] Yang D, Cao X, Wang F, Jiang H, Feng D, Guo H, Du L, Jin Y, Chen Y, Yin X, Li C (2017). LFG-500, a novel synthetic flavonoid, suppresses epithelial-mesenchymal transition in human lung adenocarcinoma cells by inhibiting NLRP3 in inflammatory microenvironment. Cancer Lett.

